# The Careers and Professional Well-Being of Women Oncologists During the COVID-19 Pandemic: Responding for Tomorrow

**DOI:** 10.2196/47784

**Published:** 2023-08-21

**Authors:** Shivani Majmudar, Stephanie L Graff, Marah Kays, Beatriz X Braz, Laurie Matt-Amaral, Merry J Markham, Ishwaria M Subbiah, Emily Bergsland, Shikha Jain

**Affiliations:** 1 College of Medicine University of Illinois at Chicago Chicago, IL United States; 2 Lifespan Cancer Institute Legorreta Cancer Center Brown University Providence, RI United States; 3 Department of Surgery University of Minnesota Minneapolis, MN United States; 4 Department of Internal Medicine Federal University of Ceará Fortaleza Brazil; 5 Department of Internal Medicine University of Miami Miami, FL United States; 6 McDowell Cancer Center Cleveland Clinic Akron General Akron, OH United States; 7 Division of Hematology & Oncology University of Florida Gainesville, FL United States; 8 Division of Cancer Medicine University of Texas MD Anderson Cancer Center Houston, TX United States; 9 Department of Medicine University of California San Francisco San Francisco, CA United States; 10 Department of Medicine University of Illinois at Chicago Chicago, IL United States

**Keywords:** oncology, women, gender equity, COVID-19, gender inequity, oncologist, health care, women physician, burnout, mental health, well-being

## Abstract

The COVID-19 pandemic exacerbated gender inequity in medicine, with women physicians reporting greater household responsibilities than their men counterparts and steeper barriers to career advancement. The pandemic highlighted the systemic assumptions and challenges faced by women physicians, which we anticipate is also true in our field of oncology. Prior literature suggests that women physicians were tasked with increased personal and professional responsibilities without compensation for their additional work, as well as derailments in career progression and significant burnout. Our aims are to highlight areas of opportunity to optimize the workplace experience of the oncology workforce and to invest in the professional well-being and sustainability of women oncologists as a step toward global workplace equity and future pandemic preparedness.

## COVID-19’s Impact on Women Oncologists

COVID-19 exacerbated gender inequity in medicine. Women physicians, despite already harboring a greater proportion of household and caregiver responsibilities, reported greater increases in household labor and childcare responsibilities than their men counterparts [1-3]. These factors resulted in women physicians having less work-life integration and steeper barriers to career advancement [4]. We anticipate that women oncologists similarly faced these challenges during the COVID-19 pandemic, impacting their clinical and academic productivity, financial stability, mental health, and career progression.

Despite the variety of clinical practice, including academia, community, or other settings (such as government, hybrid, and industry), the coveted life-work balance is difficult to achieve. More women physicians had historically modified—or were encouraged to modify—their professional careers to accommodate their personal responsibilities; this expectation holds true for all women, even those without children. The challenges of the COVID-19 pandemic had more physicians reaching for lifelines to reduce hours or limit leadership opportunities [5]. The authors previously conducted a survey of over 90 women in oncology (Multimedia Appendix 1), which revealed that the pandemic impacted the respondents’ job responsibilities and career trajectories. Overall, 34% (33/99) of women oncologists were either assigned or volunteered to perform additional clinical duties, and 20% (20/99) stated that they already had left or were exploring options to leave clinical practice due to the pandemic. Furthermore, among those with school-age children, 50% (46/93) of women oncologists noted that they incorporated remote learning into their care but did not reduce their hours to do so. Finally, in terms of mental health, 69% (63/91) reported feeling somewhat or more significantly depressed during the first 6 months (March to August 2020) of the pandemic, 87% (79/91) perceived some or more significant anxiety, and 80% (73/91) experienced moderate to significant burnout. The long-term impact of pandemic-associated career changes on women and whether differences existed between women and men (or other underrepresented groups) need additional study. However, when these adjustments are disproportionately made among women physicians, it continues to stigmatize women in medicine and threatens overall career progression [6].

## Recommendations for a Better Tomorrow

Equitable distribution of work remains paramount to career success, job satisfaction, and general workforce health, given how imbalances drive some away from the field. This includes both clinical responsibilities and academic opportunities. Prior reports suggest that as little as 12% of COVID-19–related research was authored by women [7]. Indeed, the seminal oncology paper on COVID-19 among patients with cancer included 27 women and 46 men among the authors (only 27/73, 37% women) [8]. Organizations should work to establish emergency protocols for overtime clinical coverage in case of another national health care crisis similar to the COVID-19 pandemic. There should be clear distributions of clinical time across tracks and career points in publicly available documents and established overtime pay metrics or other benefits to support the added clinical responsibilities [9]. Similarly, academic opportunity needs to be simultaneously protected for those preferring to continue their research focus within their primary research. Emerging opportunities for research on the intersection of a national health crisis and the physician’s expertise need to be considered as an open call, similar to how a new job posting or departmental grant may be considered, potentially even giving preference to junior faculty or groups who are underrepresented in medicine [8]. The responsibility to create equitable academic opportunities extends beyond academic centers and universities—it also falls to grant funders, publishers and editorial teams, and professional societies, who, as national representatives, should be champions for diversity. This would ultimately raise our collective competence and inspire innovation in our field.

Given the significant burden experienced by women oncologists and other women physicians more generally during the COVID-19 pandemic in caregiving and remote learning school responsibilities, health systems and academic centers—national leaders in care and learning—need to be positioned to support women physicians [1,4,10]. Health care facilities can partner with staff to create national crisis childcare, educational services, and older adult care support that would allow women physicians to serve in their field of expertise while providing safety for those they care for. Flexible work arrangements, emergency childcare or older adult care service benefits, childcare or older adult care marketplaces, and on-site care facilities are a few examples of successful models that would support a diverse workforce during times of national crisis.

Lastly, as the idiom says, “an ounce of prevention is worth a pound of cure.” Investing in whole-person health should be a full-time endeavor of our broader system to ensure the oncology workforce is physically and mentally fit to face a crisis. Women oncologists spent far less time on personal or self-care during the COVID-19 pandemic, which may have contributed, among many other factors, to increased anxiety, depression, and burnout [11]. Physicians should have access to an entire cadre of support services throughout their careers, which should include everything from mentorship and coaching to work-life concierge services such as the American Society of Clinical Oncology’s recent partnership with SafeHaven [12], meal preparation or delivery services, fitness services, or retreats across a spectrum of activities. Vacation and family leave policies should be reviewed and modified for optimal employee health and well-being in the modern age; this time should not only be compensated and normalized but encouraged [9].

Although further studies are warranted to determine the impacts of COVID-19 on women oncologists, we can already recognize the lasting effects on career adjustments, personal lives, and trauma. As a broader body of evidence suggests, the pandemic highlighted areas of improvement that should be prioritized among the oncology workforce; this would enable us to prepare for subsequent waves of change the future is sure to hold. The profession of medicine should allow women and men alike to share the joys of patient care and their lives equally. By investing in health today, we can ask our physicians to rise to challenges tomorrow and ensure that they will be ready.

## Appendix

Multimedia Appendix 1 Self-reported pandemic impact on women oncologists.
