# Supplementary material for: The Careers and Professional Well-Being of Women Oncologists During the COVID-19 Pandemic: Responding for Tomorrow
Source: J Med Internet Res. 2023 Aug 21;25:e47784. doi: 10.2196/47784 (PMC10477917; doi:10.2196/47784)
Supplement: Multimedia Appendix 1 [file jmir_v25i1e47784_app1.docx]

**Table S1.** Self-Reported Pandemic Impact on Women Oncologists

| Parameter | N | (%) |
| --- | --- | --- |
| **Job Responsibilities/Career** |  |  |
| Assigned or volunteered to perform additional clinical duties | 33/99 | 33.7% |
| Increased their work hours attributed chiefly to | 40/93 | 43.% |
| additional COVID-19-related professional requirements | 10/40 | 25% |
| using former commute time for work in a new remote work mode | 16/40 | 40% |
| Advanced timeline to retire or stop clinical practice | 11/94 | 11.7% |
| Career progress slowed | 51/91 | 56% |
| Pandemic negatively impacted timeline for academic promotion, time or partnership, or other career advancement metric | 31/91 | 42.8% |
| **Personal Responsibilities** |  |  |
| Accepted new caregiving responsibilities for a parent or adult not previously in their direct care | 27/99 | 27.3% |
| Incorporated virtual learning for school-aged children into their care but did not reduce their clinical hours to do so | 46/93 | 49.5% |
| Reported a noticeable reduction in take-home pay | 14/98 | 14.9% |
| **Mental Health & Well-being** |  |  |
| In the first six months of the pandemic, reported somewhat to more significant |  |  |
| Depression | 63/91 | 69.2% |
| Anxiety | 79/91 | 86.8% |
| Burnout | 73/91 | 80.2% |
| Performed less self-care than before the pandemic | 54/94 | 57.4% |
